# Intellectual stimulation in family medicine: an international qualitative study of student perceptions

**DOI:** 10.3399/bjgpopen20X101045

**Published:** 2020-06-24

**Authors:** Kiran Sahota, Patrick Goeres, Martina Kelly, Eugene Tang, Marianna Hofmeister, Hugh Alberti

**Affiliations:** 1 Faculty of Medical Sciences Cookson Building, Newcastle University, Newcastle-upon-Tyne, UK; 2 Department of Family Medicine, Cumming School of Medicine, University of Calgary, Calgary, Canada; 3 Institute of Health and Society, Newcastle University, Newcastle-upon-Tyne, UK

**Keywords:** family medicine, academic, intellectual stimulation, qualitative, focus group, undergraduate, career choice, schools, medical, general practice, primary health care

## Abstract

**Background:**

Globally, medical schools struggle to ensure there is a sufficient number of graduates choosing family medicine as a career to meet societal needs. While factors impacting career choice are complex, one possible disincentive to choosing family medicine is the perception that it is less intellectually stimulating than specialty care.

**Aim:**

The study sought to elicit student views on intellectual stimulation in family medicine, and their understanding of academic family medicine.

**Design & setting:**

This is a qualitative focus group study of volunteer students from the University of Calgary, Canada, and Newcastle University, UK.

**Method:**

Six focus groups were conducted with 51 participants. The data were analysed thematically.

**Results:**

Students associated intellectual stimulation in family medicine with clinical practice. Intellectual stimulation was related to problem solving and the challenge of having to know a little about everything, along with clinical uncertainty and the need to be vigilant to avoid missing diagnoses. Student awareness of academic family medicine was limited, and students identified it with teaching rather than research.

**Conclusion:**

Promoting intellectual stimulation in family medicine requires educators to highlight the breadth and variety of knowledge required in family medicine, as well as the need to manage clinical uncertainty and to be vigilant to avoid missing diagnoses. Exposure to academic family medicine could enhance students’ understanding and appreciation of the role of research in family medicine.

## How this fits in

While factors impacting career choice are complex, one possible disincentive to choosing family medicine is the perception that it is less intellectually stimulating than specialty care. In this study, students perceive that intellectual stimulation in family medicine is associated with clinical expertise and broad scope of practice. Promoting intellectual stimulation in family medicine requires educators to highlight the breadth of knowledge and variety of presentations available to learners. Research was associated with specialty disciplines; students knew little about research in family medicine and expressed interest in learning more, indicating the need for increased exposure to family medicine research.

## Introduction

Globally, medical schools struggle to ensure there is a sufficient number of graduates choosing family medicine as a career to meet societal needs.^1–5^ In Canada there is an expectation that at least 40% of graduates will enter family medicine,^6^ while in the UK, the Department of Health and Social Care has declared its aim to increase trainee numbers in general practice by 5000.^7^ In both countries, considerable efforts are being spent to promote family medicine as a career choice. While the factors impacting career choice are complex, one possible reason students choose hospital specialty careers over family medicine is the perception that family medicine is less intellectually stimulating than specialty care. A UK survey of medical students, at varying levels of training, found that students perceived family medicine as mundane.^8^ Students perceived family physicians as managing straightforward, simple consultations, while more complex problems were referred to hospital colleagues.^8^ These findings were supported by a recent cross-sectional survey of medical students at the University of Oxford, which found that student perception of generalist practice was associated with easy, ‘tedious’ consultations.^9^ Similarly, Australian medical students described family medicine as ‘flexible, but boring’.^10^

Such views are compounded by a well-described hidden curriculum against family medicine,^11,12^ where academically intelligent students are dissuaded from choosing family medicine owing to the perception that they will not achieve their intellectual potential. Qualitative studies of medical students also highlight students’ perceptions that family medicine is a less prestigious and academically challenging career choice.^13–15^

One potential suggestion to counter these beliefs is to promote academic family medicine; for example, by creating opportunities for research and academic mentorship.^9,16^ This includes making academic training opportunities in family medicine more accessible, ensuring all students have access to scholarly activity and supervision by family physicians, and raising the profile of senior academic family medicine leaders.^17^ The promotion of academic practice in family medicine mirrors efforts by specialty disciplines to attract students, particularly in response to concerns about recruitment.^18–20^ To date, suggestions to increase academic exposure in family medicine emanate primarily from academic family physicians. Less is known about medical students’ perspectives on the role of intellectual stimulation in family medicine and what that might mean, and their attitudes to family medicine research. This is important as most of the understanding of promoting academic support is based on studies in the postgraduate setting. A recent systematic review found few robust studies have evaluated such efforts at undergraduate level.^21^ Furthermore, there is growing concern about the decline of applicants for academic careers,^22,23^ which is even more noticeable in family medicine. In a follow-up survey of UK medical graduates, alumni of 2009 and 2012 were less likely than those of 2005 to choose academic family medicine training, with only 1.5% of graduates expressing interest in a career in academic family medicine.^24^

In this study, to tailor development of curricula that would promote family medicine as a career choice, it sought to elicit student views on intellectual stimulation in family medicine, and their understanding of academic family medicine. In addition, an understanding of student views of family medicine is of interest to practising family medicine specialists, particularly those who teach students and trainees or residents, as they reflect on their own career. The authors chose to study this question in medical schools from two similar public health-system contexts, with similar challenges in recruiting family medicine doctors, but in contrasting student contexts; for example, in the UK the majority of students are undergraduates, whereas in Canada students have already undertaken a previous degree, often with research experience, and thus may be more interested in and/or knowledgeable about academic family medicine.

## Method

### Setting

The study involved the undergraduate medical programmes of the University of Calgary, Canada, and Newcastle University, UK. The University of Calgary is a 3-year medical school. Students spend the first 2 years in a pre-clerkship phase before spending their third year in clinical clerkship. The majority of entrants have a bachelor’s degree and 22% of entrants have a graduate degree. In addition, entrants may select into an optional track to pursue a master’s or doctoral degree as part of medical school training.

Newcastle University runs a 5-year programme in which students spend the majority of their first 2 years in pre-clinical training and the following 3 years predominantly in clinical placements. The majority of students enter directly from secondary-level education. Around one-third of students select to do an additional master’s degree (intercalation) usually in academic research.

The aim of both universities is to graduate an undifferentiated physician. Thirty to forty per cent of graduates in both schools pursue a career in family medicine.

### Study design

This was a qualitative focus group study. Focus groups were chosen to encourage participants to exchange views while challenging and expanding ideas through group talk.^25^ The research team comprised two family doctors early in their training (KS and PG), a PhD medical educationalist (MH), and three academic family doctors (MK, ET, AH). With the exception of KS and PG, all co-investigators had qualitative research experience. Six focus groups were conducted between September 2018 and January 2019, three at each site.

### Sampling and recruitment

Participants comprised a convenience sample based on students’ interest in the study and availability. In Calgary, students were sent an email outlining the study through the medical school administrative office. In Newcastle, students were sent an email outlining the study through the medical school, followed by an informal invitation by a member of the research team (KS). Focus groups in both locations were timetabled during lunchtimes to facilitate student participation and students were given a snack as an incentive.

### Data collection

A semi-structured interview schedule was developed, piloted, and modified based on student feedback. Interviews were conducted by PG, MH (Canada) and KS (UK). Students gave informed consent and were identified by numbers to protect their anonymity. Focus group discussions centred on two key questions: what are your perceptions about family medicine as an intellectually stimulating career?; and what are your perceptions about family medicine as an academic career? For the purposes of this study, the definition of academic physician used by Darbyshire was adopted, which defined academic work ‘in the broadest terms, combining clinical work with scholarly work such as teaching or research’.^21^

The interview schedule is available as Appendix A. Facilitators pursued relevant themes and sought clarification or elaboration as required. Focus groups lasted between 30 and 70 minutes, and were recorded and transcribed verbatim.

### Data analysis

The data were analysed thematically.^26,27^ This involved data familiarisation, generating initial codes, and reviewing and defining themes. At each site, the local team read and coded the first focus group. Both teams met to ensure similarity in the conduct of groups, and to discuss early analysis. Each team then conducted another two focus groups at their site. The authors independently read, coded, and identified preliminary themes. This was followed by teleconference meetings to compare and contrast findings across the two sites. Subsequently, codes were reorganised and grouped, and main themes and sub-themes identified. The final themes were agreed collaboratively with discrepancies resolved until consensus was reached. The authors' developing understanding was recorded using an audit trail. Data analysis continued until a coherent interpretation of students’ views had been obtained and no new themes were identified.

## Results

### Participants

Six focus groups were conducted with 51 participants: 26 Canadian students and 25 UK students.

Students in Calgary were predominantly in the pre-clerkship phase of training (20 students), with six students in their final clerkship year. In Newcastle, all students were in the clinical phase of training, 20 in year four, five of whom were doing an intercalated degree, and five students in the final year.

### Findings

Two themes centred around the research questions: family medicine as intellectually stimulating; and students’ perceptions of family medicine and academia (see Table 1).

**Table 1.** Thematic structure, sub-themes, and codes

Theme	Sub-theme	Codes
FM as intellectually stimulating	Yes	Variety of clinical presentations
		Breadth of knowledge required
		Managing uncertainty
		Time-limited decision making (UK only)
		Diverse career opportunities
	No	The same thing every day
		Complex problems are referred to specialists
		Time-limited decision making (UK only)
		Disparaging remarks about FM as a career
		Less competitive
FM and academic practice		Views of academia in generalLack of association between research and FMAcademia in FM associated with educationLack of role modelsCuriosity to learn more about academic FM

FM = family medicine

### Theme 1: Family medicine and intellectual stimulation

Participants in both sites discussed their perceptions of family medicine as intellectually stimulating. Of note, a number of codes (Table 1) can be viewed as 'other sides of the same coin'. For example, some students valued the variety of clinical presentations in family medicine, whereas others felt that family medicine doctors see the same thing every day. Similarly, breadth of knowledge was identified as a reason why family medicine is intellectually stimulating, but the need to refer to specialists and forego in-depth management detracted from intellectual stimulation. UK students cited time-limited clinical decisions as a reason both for and against family medicine as intellectually stimulating.

#### Family medicine as an intellectually stimulating career choice

##### Variety of clinical presentations

Canadian and UK medical students attributed intellectual stimulation in family medicine to the fact that family physicians are often faced with a wide variety of presentations:

*'General practice is a very intellectual job because I think that they need to know everything from the young, to the oldest one, and whether they have any long-term conditions, or any other conditions. So, I think that a general practitioner (GP) needs a lot of knowledge about everything*.' (FG2, UK)

*'I think it is probably the most intellectually stimulating of any of the disciplines, if all I had to do was listen to hearts for the rest of my life I would die of boredom*.' (FG3, Canada)

##### Breadth of knowledge required

Some participants appreciated seeing undifferentiated illness and the need for breadth rather than just depth of knowledge:

*'I find family* [medicine] *interesting because you are often seeing very broad undifferentiated issues. When you’re the gastroenterologist … by the time they get to you, you are just managing their Crohn’s disease as opposed to figuring it out from the beginning. That’s the part I like about family medicine*.' (FG2, Canada)

*'I think of it as just as intellectually stimulating as say another, as a hospital specialty just because you have to know that breadth that I think you definitely don’t have the depth but you have certain breadth and that makes up for it*.' (FG2, UK)

The breadth of knowledge required by family physicians was also important to ensure diagnoses were not missed:

*'I feel like you have to be so on your guard at all times that you’re not going to miss something*.' (FG2, UK)

A UK participant commented on the need to 'think outside the box' when being a generalist:

*'You’ve got to be thinking that they’ve come in for this thing but is there anything else that this person needs. Like yesterday I saw someone and she thought it was going to be a really quick consultation but the GP was able to realise very quickly that she had a lot of extra things going on and knew that he needed to call her back. So, you’ve always got to be thinking outside the box not just focusing, I feel like other specialties focus on the one thing and they don’t deal with anything else it’s just like "refer on", but GPs have to deal with all of it*.' (FG3, UK)

##### Managing uncertainty

Students associated managing uncertainty, as seen in family medicine, with intellectual stimulation:

*'I think it is intellectually stimulating because you don’t know what’s coming through the door, you don’t know the age the patient’s going to be, it could be anything*.' (FG3, UK)

##### Time pressures in UK family medicine

Students in the UK commented on how time-limited consultations generated intellectual stimulation:

*'When you only have 10 minutes to see a patient I think that ramps up the intellectual stimulation so much more because you are so much more pressured*.' (FG3, UK)

##### Career flexibility in family medicine

Both student groups associated intellectual stimulation with opportunities to pursue additional interests:

*'Some GPs have special interests, like in ENT* [otolaryngology]*, or paediatrics*.' (FG3, UK)

This was not limited to specialties outside traditional family medicine but also within family medicine itself, for example, rural practice:

*'I don’t anticipate needing a lot more intellectual stimulation because the way I see family medicine you can just take so many different paths whether it be low-risk obstetrics or emergency medicine especially since I’m considering the rural route*.' (FG1, Canada)

#### Family medicine is not an intellectually stimulating career choice

Many of the reasons students gave for not finding family medicine intellectually stimulating contrasted with preceding findings.

##### The same thing every day

Both UK and Canadian medical students perceived family medicine as routine. While acknowledging the breadth of presentations, they expressed concern about the repetitive nature of seeing similar presentations:

*'It’s the desk job of medicine isn’t it? You sit in the same room and someone comes in and you do whatever and they go out. It’s just that rigmarole of the same thing every day*.' (FG3, UK)

*'What you are going to see is colds and chronic back pain and acne*.' (FG1, Canada)

##### Complex problems are referred to specialists

Although participants acknowledged the breadth of knowledge required to be a family doctor, they felt that patients with complex problems were more likely to be referred to specialty physicians:

*'All the complex cases get sent to hospital*.' (FG2, UK)

This meant that as clinicians you are not able to follow the patient through their clinical journey:

*'Basically, if anything becomes serious and you refer them on and then you’re out of the picture*.' (FG2, Canada)

##### Time-limited decision making

Some students questioned how much stimulation there could be in time-limited appointments:

*'Ten minutes, how much thinking can you do?'* (FG2, UK)

##### Disparaging remarks about family medicine as a career

Students reflected on the influence of faculty during medical school, particularly comments that diminished family practice expertise:

*'Starting points vary at entry to med school, but some enter with just a general idea of a doctor based on their personal experience, but are then influenced by hidden curriculum which implies that FPs have minimal knowledge base (only the basics), refer inappropriately (can you believe that?), and disparaging remarks by specialist*.' (FG 2, Canada)

*'From what I’ve seen just observing GPs I’m always amazed by how much they know and I just think I can’t imagine ever knowing that much. I get really angry because so many people say "oh you’re just a GP", I’ve had friends in 5th year and they are like "oh if you can’t do anything else you do GP"'*. (FG2, UK)

##### Less competitive

At the beginning of medical school, students at both sites were aware of the lack of competitiveness to become a family medicine doctor and that it was seen as a backup choice:

*'Through medical school, you know exactly where you are in the year, and then they tell you that sixty per cent of the year are going to be GPs and I think it makes it sound like that’s what people settle for, and then the very competitive, ambitious ones then become something else. That’s the way it’s been sold to us*.' (FG 1, UK)

This is problematic because of the competitive nature not only of medicine as a course but also of the individual:

*'Medical school is to keep going, keep seeing how good you can be against everybody else which is why then GP’s settling because it’s not quite as competitive*.' (FG1, UK)

Unsurprisingly then, there appeared to be a common notion that family medicine was seen as a backup career:

*'This whole idea of family *[medicine]* is the backup specialty creates a ton of issues because it gives it this lens of* *‘* *that’s the specialty you do when you can’t get into the other specialties* *’* *.* (FG3, Canada)

### Theme 2: Family medicine and academia

Medical students from both countries discussed various aspects of family medicine and academia. They did not associate research with family medicine; academic family medicine was more closely aligned with education and students described an absence of role models. Despite this, students were curious to learn more.

#### Views of academia in general

Some students appeared unclear as to the nature of academia and the role of research and/or teaching within it:

*'It does come down to the terminology by academic, what we mean by academic and I think that’s quite a vague term I think of it maybe as research based but that’s why I find it difficult to sort of come down to say what an academic career as a GP would be like because I don’t really understand the term academic, like in specifics cause it’s quite a general thing*.' (FG2, UK)

Students voiced a number of positive reasons to do academia in general, related to its potential for fun and creativity and a break from clinical work:

*'Research can be really fun if you’re passionate about it*.' (FG1, Canada)

However, more students had negative perceptions related to bureaucracy, isolation, competition, antagonist environment, and time away from patients:

*'The negative perceptions I have of it is that academia is very slow and very bureaucratic and you need to spend a lot of time writing grants and defending your ideas and sometimes it might be difficult to get things done*.' (FG3, Canada)

*'I feel like if I wanted an academic career I would be in Science, like a science degree, not a medical degree, I came for a medical degree for the clinical*.' (FG2, UK)

#### Lack of association between research and family medicine

Neither student group associated family medicine with research:

*'I wouldn’t think that there was any relationship between general practice and research*.' (FG3, UK)

*'The words “academic family medicine” seem to be used very confidently in this introduction, but I have honestly never heard those three words pieced together in that way*.' (FG2, Canada)

Underlying these perceptions seemed to be a view that research is not, or could not be, done in the family medicine setting:

*'I can’t think how it works, so would you be doing a study at the same time as being a GP?'* (FG2, UK)

*'Like I didn’t know there was family medicine research and I don’t know if they are published or if they are less accessible*.' (FG1, Canada)

Interestingly, students from both sites brought up the challenges of securing research funding in family medicine:

*'* *…* *I’m not too sure about a lot of bodies who are willing to fund a lot of research when it comes to* *g* *eneral* *p* *ractice.* *'* *(FG2, UK)*

The reason why some students could not perceive how research could be conducted in the community setting may be that it was more associated with a specialist career than a family medicine career:

*'I think specialists are really encouraged to do research so if you think of all the specialists, 90% of them do research. It’s easy to have a mentor and to be exposed to it than know about it while if you have all the family doctors and most of them don’t do research then you don’t see that as much*.' (FG2, Canada)

When students discussed family medicine research they related it to public health, sociology and, in the Canadian setting, quality improvement:

*'All I’ve ever heard GPs talk about is, social research is not the right way to put it, but it’s not in a lab, it’s more on a broad scale*.' (FG1, Canada)

#### Academia in family medicine associated with education

There was a perception from students in both sites that academia in the family medicine setting was more linked with education than research:

*'My reflex when I hear the term “academic family medicine” would be from the teaching perspective as well and possibly that’s because I don’t have a research background so that’s not where my brain reflexively goes to*.' (FG3, Canada)

*'GPs are now more involved in medical education as well. So, in a way it's more academia focused, nowadays it’s like GPs are taking on more medical education roles*.' (FG2, UK)

#### Lack of role models in academic family medicine

The lack of association of family medicine with academia could be attributed to the lack of visibility of those in academic positions. Students described a lack of family medicine academic role models as a stumbling block:

*'I don’t think I’ve seen as many GPs who have been involved in research, at university I feel like quite a lot of our lecturers are specialists and you don’t see quite as many GPs*.' (FG2, UK)

*'I guess one thought that I have had over the past few years really is I haven’t had much exposure to mentors who have done the career in academic family medicine in particular. So, I think if I got to see what that career would look like and maybe get a chance to speak to someone who was prolific in terms of their scientific career as well in family medicine I think that would be super interesting*.' (FG1, Canada)

#### Curiosity to learn more about academic family medicine

Finally, students seemed keen to know more about the possibilities of academic work within the family medicine setting:

*'From what I’ve seen so far,* [family medicine is] *way different than what I expected … I didn’t know you could do research as a family doctor. So, this is something new I think it’s important for people to know*.' (FG1, Canada)

*'I’ve realised there is a bit more academic in GP than I thought. It seems more bearable than lab-based academic stuff*.' (FG2, UK)

## Discussion

### Summary

In this novel international study, it was found that students in both Canada and the UK associated intellectual stimulation in family medicine with clinical practice. Intellectual stimulation was associated with problem-solving and the challenge of having to know a little about everything. It was also associated with clinical uncertainty and the need to be vigilant to avoid missing diagnoses. In the UK, this was compounded by the challenge of time-limited consultations. For some learners, the breadth of knowledge required was off-putting. These students preferred to know a topic in depth and were frustrated at the thought of having to refer a patient to specialty care for further management. Overall, student awareness of academic family medicine was limited, associated primarily with teaching rather than research. The notion of family medicine research was relatively novel and students struggled to identify a role for family physicians as researchers; what topics they would research, and what methodologies they would use? Research was associated with specialty practice.

### Strengths and limitations

This is the first study to the authors' knowledge that has explicitly focused on students’ perceptions of family medicine and academia or intellectual stimulation. The consistent findings across two international sites, with the notable exception of the influence of the consultation time, suggest that the findings may well be transferable to other settings in the UK and Canada, and other countries with similar healthcare systems.

The findings represent the opinions of a self-selected group of students and may not be generalisable to the entire cohort of learners at the authors' respective institutions, nor other schools. The authors recognise that the context of the schools is somewhat different, but also note that in both countries family medicine is publicly funded and promoted by governments as a key ingredient in the delivery of high quality patient care. The focus group setting may have inhibited some students from sharing their opinions. However, the focus group setting generated discussion among students and the open-ended questions resulted in descriptions of experiences and suggestions for improvement that had not been considered. Detailed demographic data on the participants in not known, nor is their current career preference; however, as an explorative study, this was not felt to be essential.

### Comparison with existing literature

These findings correlate with previous research published on students’ perceptions of family medicine. The finding that students associate intellectual stimulation with clinical practice emphasises the central role of high-quality clinical placements and preceptor role modeling in promoting family medicine.^28,29^ These data continue to speak of the denigration of family medicine during medical school and confirm previous findings.^11,12^

### Implications for practice and research

 Students’ association of intellectual stimulation with clinical practice could be harnessed to make core tenants of family medicine practice more overt. This includes teaching students strategies to assess patients presenting with undifferentiated symptoms, use of safety netting to mitigate missing potentially serious diagnoses, and how family physicians negotiate and manage time. The findings also suggest potential opportunities to address student misconceptions; for example, by discussing with students the variability of ‘routine’ presentations by comparing and contrasting two similar presentations. Preceptors could also discuss how application of evidence-based practice to seemingly simple problems, such as a sore throat and back pain, continue to challenge health systems. Students could be given opportunities to reflect on complex case presentations; for example, by emphasising the role of multimorbidity in family medicine and that despite short respites in hospital, there is robust evidence for the importance of continuity.^30^

Denigration of family medicine during medical school appears to be continuing:^11,12^ a number of participants noted that specialist lecturers often referred to the minimal knowledge base required of family physicians and unnecessary referrals based on their perception of mismanagement on behalf of the family medicine doctors. These findings indicate that the challenge of promoting intellectual stimulation in family medicine extend beyond departments of family medicine, and require initiatives and ongoing monitoring at faculty level. The call for increased respect across specialties within medicine is echoed by the study authors, in particular across the primary—secondary care divide, and it is recommended that clinical family medicine specialists be careful not to denigrate their own specialty.^11,12^

Students associated research with specialty practice. They struggled to identify what type of research family physicians could do, thinking primarily of audit and quality improvement or small-scale studies on community needs. Students also felt family physicians would be less competitive when applying for research grant funding. A number of participants volunteered for the focus group study in order to learn more about academic family medicine. Students in both countries were curious to learn more about family medicine research, supporting recent initiatives.^17^ Strategies to achieve this could include ensuring family medicine research is referenced within course material,^31^ and giving opportunities for students to interact with family medicine researchers through lectures, research electives, mentorship, or development of specific family medicine research modules. An important caveat from the study is that overpromotion of academic family medicine could, however, be a deterrent to a career in family medicine. Some students expressed that they were specifically interested in family medicine as they would not have to engage in research. Some learners, particularly Canadian students with research experience, found the research environment competitive and isolating, and commented that having to do research would be a disincentive to selecting a career in family medicine. This is in keeping with previous surveys on careers in family medicine in the UK^2^ and may ring true with clinical family medicine doctors who, like some students, do not have an active interest in research.

In the international study, medical students associated the presence or absence of intellectual stimulation in family medicine with clinical practice. Their knowledge of academic family medicine was limited and primarily associated with teaching rather than research. They were keen to know more and it is suggested that increased exposure to academic family medicine and research could enhance students’ understanding and appreciation of the role of intellectual stimulation, and maintenance and refreshment of long-term goals.
